# Screening for Melanoma and Other Skin Cancer Shows a Higher Early Melanoma Incidence: Social Educational Program “Life Fear-Free”

**DOI:** 10.3390/dermatopathology8010011

**Published:** 2021-03-15

**Authors:** Lev Demidov, Igor Samoylenko, Nina Vand, Igor Utyashev, Irina Shubina, Igor Sinelnikov

**Affiliations:** 1 FSBI N.N. Blokhin National Medical Research Center of Oncology, Ministry of Health of Russia, Moscow 115548, Russia; demidov.lev@gmail.com (L.D.); dr.utyashev@gmail.com (I.U.); 2 Russian Melanoma Professional Association (Melanoma.PRO), Moscow 119192, Russia; nina.s.vand@gmail.com (N.V.); sinelnikov.igor@gmail.com (I.S.)

**Keywords:** cutaneous melanoma, melanoma screening, skin cancers, social and educational program

## Abstract

Background: The screening program Life Fear-Free (LFF) aimed at early diagnosis of cutaneous melanoma (CM) was introduced in Samara, Chelyabinsk, Yekaterinburg, and Krasnodar (Russia) in 2019. Objectives: To analyze the impact of the program on early CM and non-melanoma skin cancer (NMSC) detection. Methods: According to the social educational campaign, people were informed about CM risk factors and symptoms and were invited for skin examination. The program planned to involve 3200 participants in total. Participants with suspicious lesions were invited for excisional biopsy. Results: 3143 participants, including 75.4% women, were examined for skin lesions. The average age of the participants was 43.7 years. Mostly skin phototypes II and III were registered (48.2% and 41.0%, respectively); 3 patients had CM, 15 had basal cell carcinoma, and 1 had Bowen’s disease, which were confirmed histologically. All detected melanomas had Breslow’s thickness of 1 mm. Conclusion: The participants showed high interest in early skin cancer detection programs. The incidence rate of CM and NMSCs among the program participants was higher than in general public. The early disease grade was proven for the detected CMs and NMSCs. The study has shown that it is important to continue such programs.

## 1. Introduction

According to World Health Organization statistical data (WHO, 2018), cancer is one of the major causes of death worldwide, with skin cancers holding the fifth rank, and cutaneous melanoma (CM) causing most deaths of skin cancers [1]. Timely referral to the doctor helps early detection and removal of thinner CMs (<1 mm), which leads to positive outcomes for the patients [2]. Thus, early CM diagnosis is an essential target for national healthcare.

An earlier epidemiological study showed that the average melanoma thickness accounted for >4 mm at the first medical examination in Russia [3]. A study in 2018 revealed several problems, such as: people were almost unaware of the disease; procedures for excisional biopsy were too complicated; insufficient special training of primary care personnel (in 2018, CM was detected in 31.9% of patients, comparable with 45.5% of patients with detected skin cancer [1]). Pathological CM overdiagnosis or underdiagnosis have become a frequent problem worldwide including Russia [4]. Each of these factors ultimately affects the final data of the early detected melanomas and the mortality rate from CM.

The Melanoma Professional Association (MPA) supported by BIOCAD (Biotech company, Russia) designed a social educational program “Life Fear-Free” (LFF) for early CM detection. The major goal was to evaluate the impact of the LFF program on early CM and non-melanoma skin cancer (NMSC) detection. In addition, the participants’ answers should provide the data for population awareness about skin melanoma, its risk factors, prevention, and early diagnosis. The program was implemented in four Russian cities in 2019.

## 2. Materials and Methods

### 2.1. Program Participants

The only inclusion criterion for the program participants was age limit—adults over 18. The cities of Samara, Chelyabinsk, Yekaterinburg, and Krasnodar, whose residents were involved in the Program, were chosen based on the CM incidence rate, population >1 million, and regional healthcare support. The LFF program enrolled 3143 participants. The number of female participants was significantly higher than male participants, including 2369 women (75.4%) and 774 men (24.6%). Such a proportion was almost the same in all studied regions. The average age accounted for 43.7 (95% confidence interval (CI): 43.2–44.3) years. However, the age difference was registered as following: the average age of 44.7 (95% CI: 43.6–45.7) and 45.1 (95% CI: 44.2–45.9) in Samara and Chelyabinsk, respectively, was higher than that of Yekaterinburg, and Krasnodar, 41.6 (95% CI: 40.4–42.8) and 42.1 (95% CI: 40.6–43.6), respectively.

The analysis also included historical data received after program LFF completion.

### 2.2. Analysis of Melanoma Awareness

Prior to the start of the screening program, an information and communication campaign was carried out in the involved cities. The campaign lasted for 2–4 weeks and included:

- design and launch of information and educational “long-read” websites about melanoma and the importance of its early diagnosis;
- launch of advertising and informational videos on TV;
- launch of advertising and information programs on the radio;
- presentations of leading oncologists and/or dermatologists of the region on TV;
- announcement of the LFF program on the regional news channels and information websites;
- announcement of the LFF program in social networks;
- contextual advertising; and
- information in bloggers’ posts.

At the first stage of the LFF program, a sociological study assessed participants’ melanoma awareness and their willingness for self-monitoring their moles or visiting a medical unit for lesion monitoring. The survey involved 1600 respondents who answered by phone. The interviews were conducted in accordance with the stratified dual-frame random sample selection [5].

### 2.3. Examinations

The participants made an appointment with the doctor via special website or by phone. All participants gave their written informed consent for inclusion before they participated in the study. The study was conducted in accordance with the Declaration of Helsinki, and the program was approved by the Ethics Committee of the Federal State Budgetary Institution “N.N. Blokhin National Medical Research Center of Oncology” of the Ministry of Health of Russia (Program approval of 5.08. 2019).

Skin examinations were performed in a two-stage mode. In Samara and Yekaterinburg, dermatologists/venereologists completed the first-stage skin examination by a dermatoscope. In Chelyabinsk and Krasnodar, oncologists performed the first-stage skin examination with or without dermatoscopes. In case of a suspicious lesion, the participant went for the second-stage examination visiting an oncologist/oncodermatologist.

**Figure 1:**
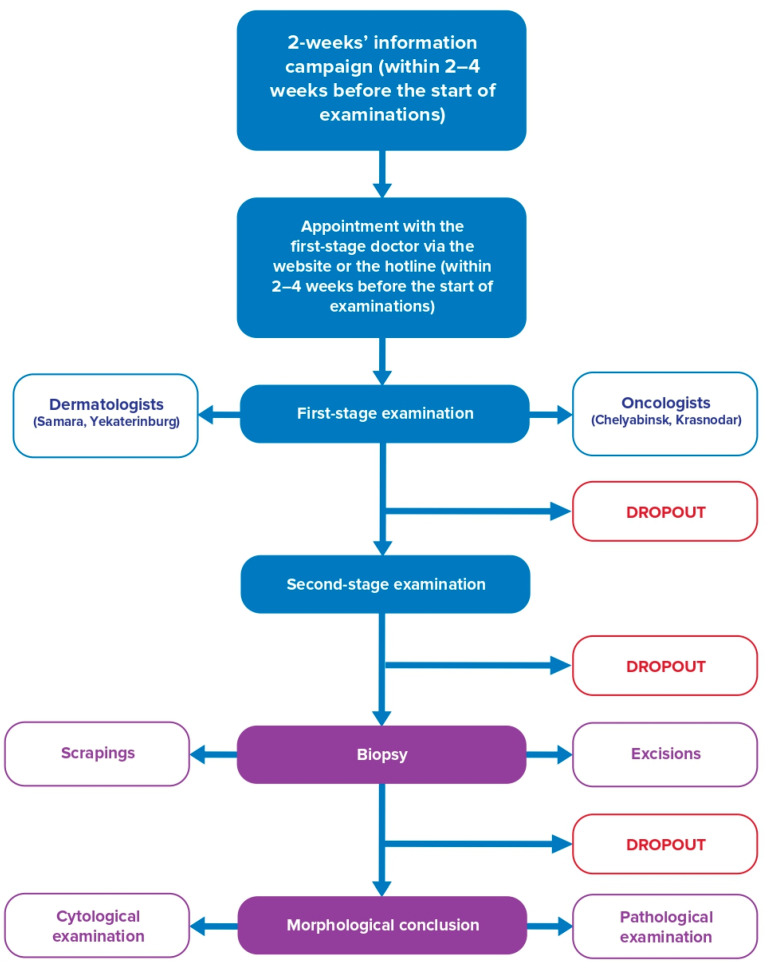
The flow chart of the study of the program “Life Fear-Free”.

All participants filled in a questionnaire concerning CM risk factors. When the participant visited the medical unit, the doctor evaluated the skin phototype and the number of nevi, and reported of any suspicious lesion in a special card in the questionnaire. The doctor classified all skin lesions as following: (1) Suspicion of melanoma or skin cancer; (2) healthy, with a high risk of skin cancer OR benign lesions; or (3) healthy. No further consultation is required. The organization of the second-stage examination was similar to the first one and finally, the doctor decided whether a skin biopsy was required. The biopsy procedure was then organized in accordance with the local practice (Figure 1).

### 2.4. Statistics

According to the program, the number of participants should be 3200 across four regions (800 per city). The collected anonymized data were analyzed by descriptive statistics.

## 3. Results

### 3.1. Awareness of the Population about Early Detection of Skin Cancer

The survey results showed that a high percentage of respondents had incorrect understanding about melanoma and skin cancer, their risk factors, and treatment methods.

Approximately 57% of the respondents either never heard the word “melanoma” (36%) or could not explain the meaning (21%). However, only 25% (out of 43% of respondents who claimed they could explain the meaning of the term) were able to describe correctly the term melanoma as a “malignant skin lesion”. Only 31% of respondents associated the development of melanoma with a sunburn, while 38% were convinced that melanoma resulted from a mole trauma. Approximately 52% of the respondents were sure that bleeding of the “mole” was the only reason why they should consult a doctor [5]. The LFF program included preparation and distribution of the information and educational materials among the participants. The materials consisted of posters and leaflets with patient-oriented pictures and texts, explaining melanoma and skin cancer features, what may cause them, how you should monitor your moles, and how to take medical examination [6]. The effect of distribution of these materials in terms of melanoma awareness has not been studied, yet.

### 3.2. Organization of Skin Examination

In total, 3143 participants from four regions of Russia took part in the LFF program from 15 October 2019 to 14 December 2019.

The study period in four regions varied according to the preferences of the regional medical organizations (Table 1).

### 3.3. Evaluation of Risk Factors for CM

In general, the distribution of risk factors related to CM and NMSC development was similar in four regions. The paper presents the data of the total population involved in the study. On the whole, 2369 women (75.4%) and 774 men (24.6%) participated in the program.

**Figure 2:**
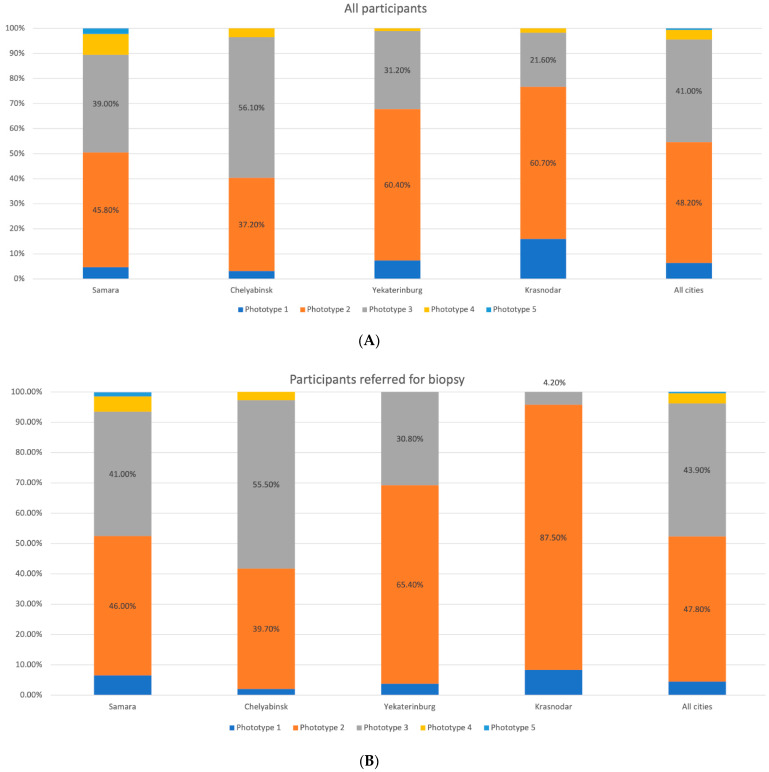
Distribution of the participants by skin phototype in the studied regions. (**A**) All participants. (**B**) Participants with suspected melanoma or other skin cancers.

**Figure 3:**
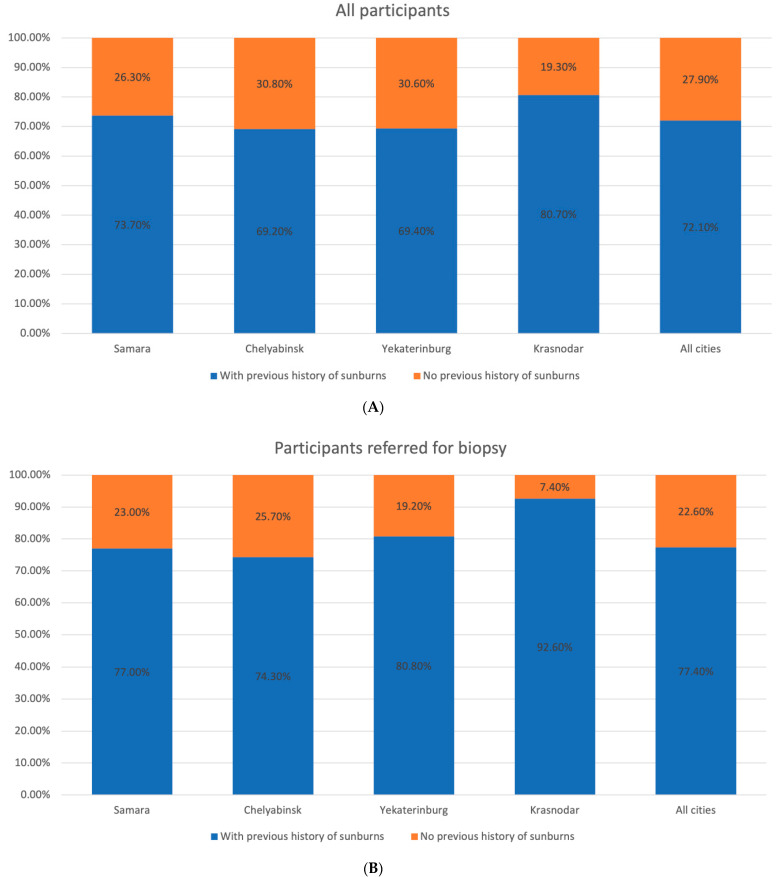
Distribution of the participants by previous sunburns in the studied regions. (**A**) All participants. (**B**) Participants with suspected melanoma or other skin cancers.

**Figure 4:**
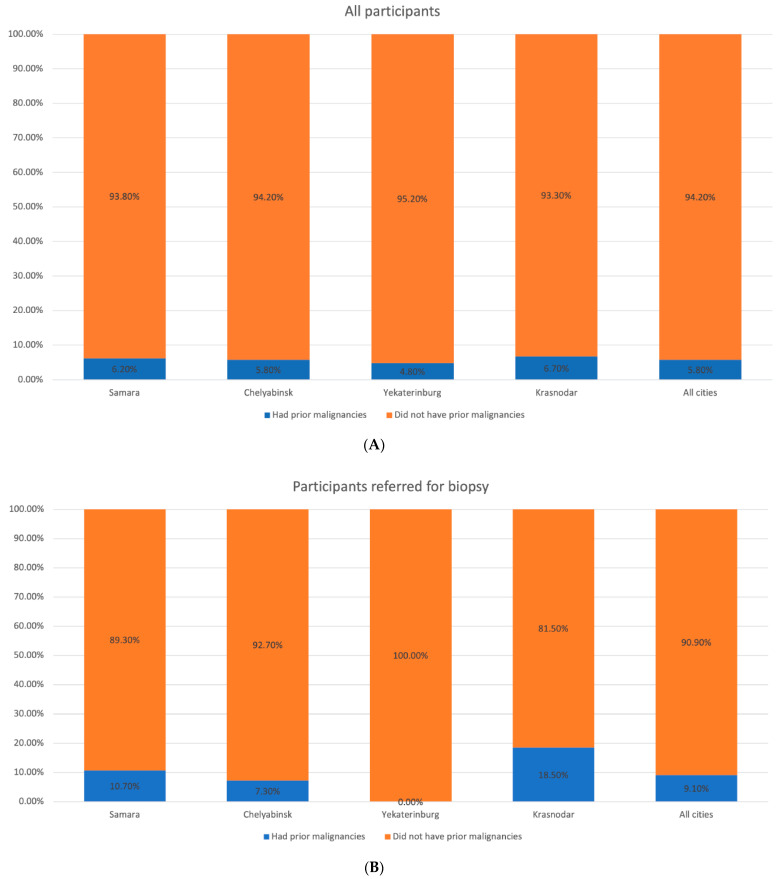
Distribution of the participants by the cancer in their personal history in the studied regions. (**A**) All participants. (**B**) Participants with suspected melanoma or other skin cancers.

**Figure 5:**
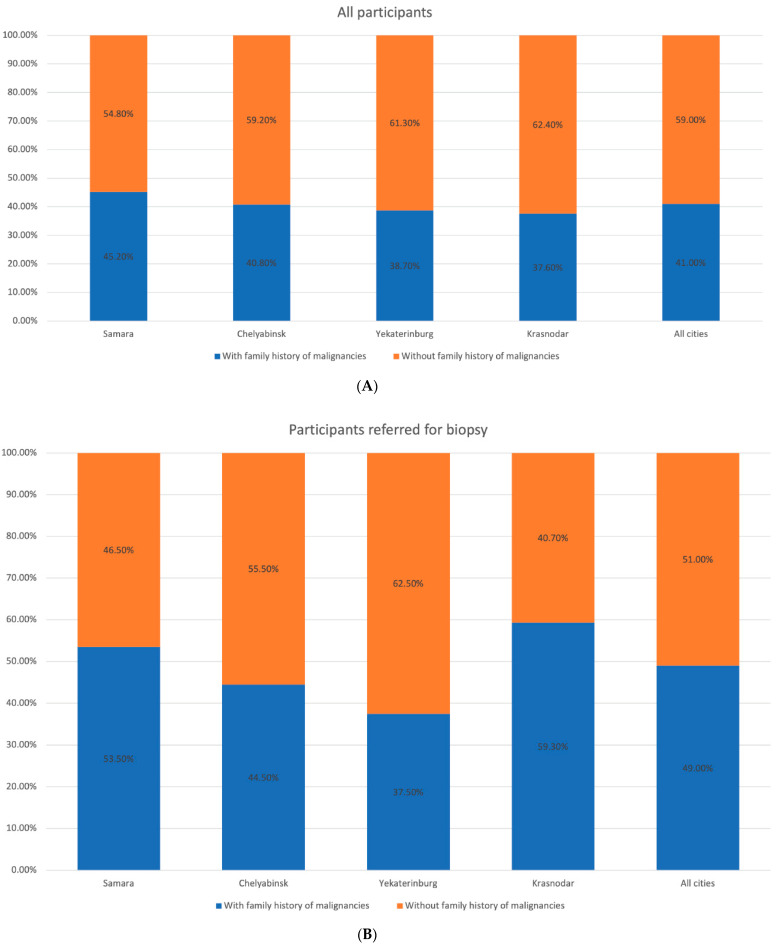
Distribution of the participants by the malignancies in the family history in the studied regions. (**A**) All participants. (**B**) Participants with suspected melanoma or other skin cancers.

**Figure 6:**
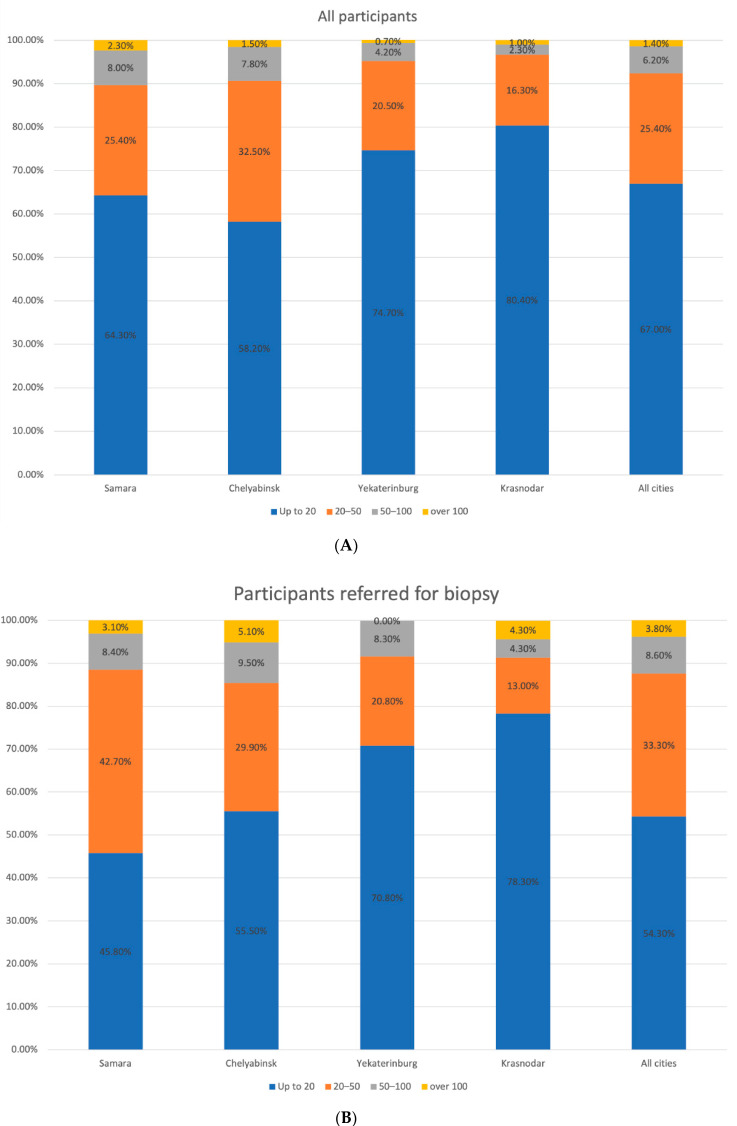
Distribution of the participants by the skin nevi in the studied regions. (**A**) All participants. (**B**) Participants with suspected melanoma or other skin cancers.

At the first stage, mostly participants with Fitzpatrick skin phototype II and III (48.2% and 41.0%, respectively) were enrolled in the study, while at the second stage the number of people with skin phototype I and II tended to rise. We observed a similar trend in the population with a large number of skin nevi, previous history of sunburns, and malignancies in their personal or family history (Figure 2, Figure 3, Figure 4, Figure 5 and Figure 6).

**Figure 7:**
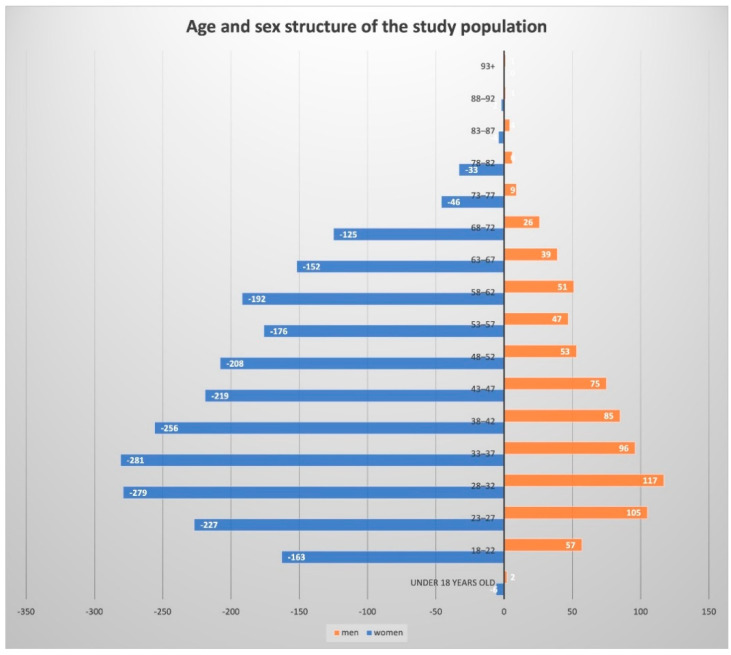
Distribution of the participants by age and sex.

The average age of the participants was 43.7 (95% confidence interval (CI): 43.2–44.3) years. Figure 7 presents participant distribution by age and sex enrolled in the social educational LFF program.

### 3.4. CM and NMSC Incidence

3 people of 3143 participants had CM, 15—basal cell carcinoma (BCC), and 1—squamous cell carcinoma in situ (SCC). CM was detected in two men (aged 40 and 72) and one woman (aged 68). The participants with confirmed melanoma had less than 20 nevi. One person had skin phototype I, 1—phototype II, and 1—phototype III. NMSC was detected in 5 men and 11 women (average age 63.6 and 69.1 years, respectively). Moreover, 11 of 16 participants (69%) had up to 20 nevi. Almost 69% of participants with confirmed NMSC had skin phototype II, 31% had skin phototype III, and 88% (14 people) had a previous history of sunburn. Two of sixteen patients had concomitant cancer, one had BCC. The characteristics of participants diagnosed with melanoma or NMSC are shown in Table 2.

A total of 68 biopsy procedures were performed (including 58 excisional biopsies with pathological examination, and 10 scrapings or smears with cytological examination), see Table 3.

Three (100%) patients with CM had Breslow’s depth of 1 mm (which corresponds to stage T1b and stage I).

## 4. Discussion

Although screening high-risk patients for melanoma has been a hot issue over the decades, no randomized controlled trials have been presented and therefore, we cannot evaluate the effect of screening on disease-related mortality.

The most extensive studies demonstrated controversial results of CM screening [7,8,9,10,11,12,13,14,15,16].

A two-stage program on melanoma and non-melanoma skin cancer (SCREEN) was introduced in Schleswig-Holstein, Germany, in 2003. Approximately 19% of the regional population participated in the program. The results showed an increase in invasive melanoma incidence by 34% among the SCREEN participants. Five years after the program completion, the researchers registered a significant reduction of the melanoma-related mortality. The authors of the program admitted they could not arrange a randomized controlled trial [17]. The SCREEN results boosted a skin cancer screening program across Germany, however no decrease in melanoma-related mortality was registered at the federal level [18].

A large melanoma screening program (1984–1996) involved the employees of the Lawrence Livermore National Laboratory in the United States. Any participant who detected a suspicious lesion during skin self-examination went for specialist’s medical examination. Participants with melanoma, as well as individuals of the high-risk group, underwent planned examinations every 3–24 months. The study showed a reduction in the rough incidence rate of melanoma with a depth of > 0.75 mm (22.1–4.62 cases per 100,000 for the study period and 15.13–4.62 cases per 100,000 during the screening period). Though the estimated expected mortality rate was 3.39, no melanoma-related deaths occurred over the observation period. The authors declared some correlation between the decrease in melanoma-related mortality and decrease in the incidence of mature melanoma (depth > 0.75 mm), the increase in the awareness of the studied population, the introduction of skin self-examination, and screening examinations for people at high risk. However, that study was neither randomized, nor controlled [19].

Finally, a large randomized study of melanoma screening started in Australia. Phase I lasted 18 months and involved 18 community-based populations (the study arm—9 communities, and the control arm—9). The number of participants in the screening study arm was higher than that of the control arm; however, the study was discontinued due to funding problems [20].

In addition, numerous reports describe social educational projects, such as Euromelanoma Day [21]. The present LFF program seems very much similar to the latter in terms of its organization.

The results of the LFF program showed that the studied population had low awareness of CM and NMSC and their risk factors. Although the program included information and awareness-raising campaign, the study did not evaluate the follow up effect of the distributed educational materials. However, a 2-week information campaign involving various media tools ensured a large number of people participated in the program (3134 enrolled participants, with initially planned 3200).

As a result of active screening (i.e., invitation for skin examinations), the researchers could pool the patients and improve CM detection that reached 3 cases of 3143 participants. The result corresponds to the incidence of 95.45 per 100,000 people, while an average Russian rough incidence rate is 7.76 cases per 100,000 adult population; thus, the study results showed 12.3 times higher incidence than the average incidence rate in Russia. NMSC incidence data showed similar result.

Apparently, given such a small number of participants, the program could not ensure any essential effect on CM or NMSC-related mortality in the participating regions.

The age characteristics of the program participants under screening for melanoma differed significantly from the average age of CM patients at the time of diagnosis; in Russia, the average age of the first detected CM is 61.7 years [1]. The Russian population with first detected NMSC is even older: the average age of patients with NMSC is 69.7 at the time of diagnosis [1].

We believe that the younger age of examined program participants might have some impact on the CM and NMSC detection results. The CM detection rate in the studied population reached 0.10% (3 patients of 3143 participants), and the detection rate of NMSCs (BCC and SCC) accounted for 0.51% (16 patients out of 3143 participants).

We compared CM incidence registered in LFF program with the incidence in the Euromelanoma Day in Sweden, 2008 [21]. A total of 24 patients of 2799 examined in Sweden had histologically confirmed CMs; therefore, the detection rate accounted for 0.9% [14]. The available data of the Euromelanoma Day project introduced in 2009–2010 in 20 European countries showed that Sweden had the highest melanoma detection rate among the countries included in the analysis [15].

Several factors may contribute to the lower CM detection rate in the LFF program. Firstly, the CM incidence in Sweden is significantly higher than that in Russia. The standardized incidence rates in Sweden were 23.5 in men and 26.2 in women per 100,000 in 2018 [22]; while in Russia, the standardized incidence rates were 4.57 in men and 4.97 in women [1].

Secondly, the average age of participants in the Swedish Euromelanoma Day was higher than that in the Russian LFF Program (53 and 43.7 years, respectively). During the Euromelanoma Day in Sweden, patients were charged for their visits, which could prevent people with no suspicion for skin cancers from participation in the project. The participants of Euromelanoma Day who had suspicious lesions underwent excision surgery on the examination day, which reduced the risk of missing histological confirmation of the diagnosis as a result of the participant’s non-appearance for the biopsy after examination. Thus, some participants of the LFF program with suspected melanoma or non-melanoma skin cancer, who had to undergo a biopsy, did not turn up for that appointment (25 people).

To increase the program effectiveness, the awareness-raising campaign should focus on the people at high risk (older people with sun-damaged skin) and therefore, the authors should analyze and adapt the information campaign.

We consider it important to pursue certain organizing measures to avoid “losses” during the inter-stage routing of the participants (initial examination—biopsy). One such measure is performing biopsy on the day of the examination.

In addition, the proficiency of dermatologists, oncologists, and pathologists in detecting skin malignancies remains one of the key factors. Possible misdiagnosis, such as incorrect phototypes or underestimated number of nevi, etc., should be minimized.

The improvement of these factors combined with administrative support can ensure the start of a CM screening program in different regions of the country.

## 5. Conclusions

The participants showed high interest in early skin cancer detection program. However, the study revealed that a high percentage of LFF program participants had an incorrect understanding of melanoma and skin cancer, their risk factors, and treatment methods.

The detectability of melanoma and non-melanoma skin cancers is comparable to that of skin cancers obtained in similar screening projects. The early stage disease was confirmed for the detected melanoma and skin cancer. The incidence rate of CM and NMSCs among the program participants was higher than in general public.

The results suggest that social educational programs should be continued and target high-risk population. The implementation of the program also demonstrated what should be improved in the campaign, and it revealed the need for high-qualified medical personnel. Firstly, the design and organizational efforts should focus on the higher risk groups to reach senior members of the society. Additionally, it appeared important to improve the patient routing to avoid discrepancies between the examination stage and biopsy completion so that, if necessary, biopsy could be performed on the examination day. Furthermore, the electronic system of collecting the examination results should be upgraded. Finally, the involved specialists, such as dermatologists, oncologists, and pathologists should have appropriate qualification and motivation to participate in such programs.

Further well-organized controlled studies are required to evaluate if CM screening could decrease melanoma mortality rate.

## Figures and Tables

**Table 1:** Organization of the social educational program “Life Fear-Free” aimed at the early detection of melanoma and skin cancers.

	Samara	Chelyabinsk	Yekaterinburg	Krasnodar	Total
Information and communication campaign start date	5 October 2019	8 October 2019	12 November 2019	26 November 2019	-
Duration of the information and communication campaign before the start of the examinations (days)	11 days	18 days	18 days	18 days	-
Start and end dates for examination appointment	5–14 October 2019	2 October ^1^–22 October 2019	8 October ^1^–2 December 2019	23 November ^1^–7 December 2019	-
Start date for first-stage examinations	15 October 2019	26 October 2019	3 December 2019	14 December 2019	-
Duration of first-stage examinations (days)	4 days (15–18 October)	2 days (26–27 October)	5 days (3–7 December)	1 day (14 December)	12
Start date for second-stage examinations	16 October 2019	26 October 2019	7 December 2019	14 December 2019	-
Duration of second-stage examinations (days)	4 days (16–19 October)	2 days (26–27 October)	6 days (7 December, 9–13 December)	1 day (14 December)	13

^1^ The participants started making appointments before the information and communication campaign since the appointment option was open on the program website before the start of the information and communication campaign.

**Table 2:** Characteristics of the participants with melanoma or non-melanoma skin cancer detected in the LFF program.

City	Sex (M/F)	Age (Year of Birth)	Number of Nevi	Skin Photo Type	Sunburns	Previous Cancer in Personal History	Previous Cancer in Family History	Diagnosis	Depth of the Lesion
Yekaterinburg	M	72 (1947)	Up to 20	II	No	No	No	Melanoma	Up to 1 mm
Chelyabinsk	M	40 (1979)	Up to 20	III	Yes	No	Yes (father, gastric cancer)	Melanoma	1 mm
Samara	F	68 (1951)	Up to 20	I	Yes	No	No	Melanoma	1 mm
Krasnodar	F	73 (1946)	Up to 20	II	Yes	No	Yes (father, lung cancer)	Basal cell carcinoma	
Krasnodar	F	72 (1947)	Up to 20	II	Yes	No	Yes (mother, skin cancer)	Basal cell carcinoma	
Krasnodar	M	71 (1947)	Up to 20	II	Yes	Yes (prostate cancer)	No	Bowen’s disease	
Yekaterinburg	F	68 (1951)	Up to 20	II	Yes	No	No	Basal cell carcinoma	
Yekaterinburg	F	66 (1953)	Up to 20	II	Yes	No	No	Basal cell carcinoma	
Yekaterinburg	M	68 (1951)	Up to 20	III	Yes	No	Yes (father, mother)	Basal cell carcinoma	
Yekaterinburg	F	81 (1938)	Up to 20	II	Yes	No	Yes (mother, uterine cancer)	Basal cell carcinoma	
Yekaterinburg	F	83 (1936)	Up to 20	II	Yes	No	Yes (mother: lung cancer, sister: ovarian cancer)	Basal cell carcinoma	
Yekaterinburg	F	56 (1963)	20–50	II	Yes	No	Yes (mother)	Basal cell carcinoma	
Chelyabinsk	M	51 (1968)	50–100	III	Yes	No	Yes (father, gastric cancer)	Basal cell carcinoma	
Chelyabinsk	M	48 (1970)	50–100	II	Yes	No	Yes	Basal cell carcinoma	
Chelyabinsk	M	80 (1939)	Up to 20	II	No	No	No	Basal cell carcinoma	
Chelyabinsk	F	71 (1948)	Up to 20	III	Yes	No	No	Basal cell carcinoma	
Samara	F	70 (1949)	Up to 20	III	No	No	No	Basal cell carcinoma	
Samara	F	61 (1958)	Over 100	III	Yes	No	No	Basal cell carcinoma	
Samara	F	59 (1960)	20–50	II	Yes	Yes (basal cell carcinoma)	No data	Basal cell carcinoma	

**Table 3:** Skin lesion biopsies performed after skin examinations of the LFF program participants.

Biopsy Results				
		Absolute Number	Percentage of All Participants (*n* = 3143)	Percentage of Participants Who were Referred for Biopsy/Underwent Biopsy without Referral (*n* = 100) ^1^
Participants referred for biopsy:	Non-informative	11	0.3	11.0
	Melanocytic nevus	23	0.7	23.0
	Other benign skin lesions	3	0.1	3.0
	Hemangioma	1	0.0	1.0
	Seborrheic keratosis	5	0.2	5.0
	Inflammatory skin diseases	3	0.1	3.0
	Sebaceous hyperplasia	2	0.1	2.0
	Ulcer	1	0.0	1.0
	Skin melanoma	3	0.1	3.0
	Skin cancer	16	0.5	16.0
	Did not have biopsy performed	5	0.2	5.0
	Did not attend a biopsy appointment	27	0.9	27.0
	Total	100	3.2	100.0
Participants not referred for biopsy:		3043	96.8	
Total		3143	100.0	

^1^ Seven participants underwent biopsy in Samara, including 3 persons who received referral “Healthy. No further consultation required” after examination by the oncologist; 3 who received referral “Healthy, with a high risk of skin cancer, benign lesions. Follow up by a dermatologist recommended” after examination by the oncologist; and 1 who did not attend the second-stage examination by the oncologist or had no data available at the second-stage examination by the oncologist.
